# Beta-lactam hypersensitivity: not always what it seems

**DOI:** 10.1186/1939-4551-8-S1-A168

**Published:** 2015-04-08

**Authors:** Djanira Andrade, Katia Jojima, Alex Lacerda, Ligia Maria Oliveira Machado, Luis Felipe Ensina, Inês Cristina Camelo Nunes, Dirceu Sole

**Affiliations:** 1 Federal Universtity of Sao Paulo, Brazil; 2 Brazilian Society, Brazil

## Background

Beta-lactam allergy is a frequent cause of visit to the allergist office. The aim of this study was to describe the characteristics of children with a suspected beta-lactam hypersensitivity reaction in a specialized drug allergy unit.

## Methods

Retrospective analysis based on medical records using an adapted ENDA questionnaire of patients under 18 years old from July 2011 to June 2014.

## Results

One hundred and four children were evaluated with a suspected drug allergy history, with 28% reporting reactions to beta-lactam antibiotics. The mean age was 6.2 years and 52% were female. Cutaneous symptoms were the most frequent reported (89%), followed by respiratory (45%). Most of them had maculopapular exanthema (52%). Urticaria and/or angioedema were seen in 34% of patients. The majority of the reactions were mild/moderate (93%), occurring in the first 24 hours after drug intake (77%), and 48% presented associated fever. The suspected drugs were: amoxicilin (59%), cefalexin (16%), penicilin and ceftriaxone (8% each). Patients went to an Emergency Unit in 97% of the reactions and treated with anti-histaminic drugs and corticosteroids in 40% and 30% respectively. Epinephrine was used in just one patient. In almost half of the patients the clinical history was not consistent enough to submit them to an extensive investigation. Of those who were investigated, skin tests were performed in 48% (57% prick tests and 43% intradermal tests). Positive test was seen in only one patient (cefazolin). Drug provocation tests with amoxicilin were performed in 57% of patients and none was positive.

## Conclusions

The prevalence of children with a beta-lactam hypersensitivity history is high, but a few cases are confirmed as allergic after an adequate investigation.

